# Study of the response of the penile corporal tissue and cavernosus muscles to micturition

**DOI:** 10.1186/1471-2490-8-4

**Published:** 2008-03-02

**Authors:** Ahmed Shafik, Ismail A Shafik, Olfat El Sibai, Ali A Shafik

**Affiliations:** 1 Department of Surgery and Experimental Research, Faculty of Medicine, Cairo University, Cairo, Egypt; 2 Department of Surgery, Faculty of Medicine, Menoufia University, Shebin El-Kom, Egypt

## Abstract

**Background:**

The reaction of the corpora cavernosa (CC), the corpus spongiosum (CS), the bulbocavernosus (BCM) and ischiocavernosus (ICM) muscles to passage of urine through the urethra during micturition is not known. We investigated the hypothesis that the passage of urine through the urethra stimulates the corporal tissue and cavernosus muscles.

**Methods:**

In 30 healthy men (mean age 42.8 ± 11.7 years), the electromyographic activity (EMG) of the CC, CS, BCM, and ICM were recorded before and during micturition, and on interruption of and straining during micturition. These tests were repeated after individual anesthetization of urethra, corporal tissue, and cavernosus muscles.

**Results:**

During micturition, the slow wave variables (frequency, amplitude, conduction velocity) of the CC and CS decreased while the motor unit action potentials of the BCM and ICM increased; these EMG changes were mild and returned to the basal values on interruption or termination of micturition. Micturition after individual anesthetization of urethra, corporal tissue and cavernosal muscles did not effect significant EMG changes in these structures, while saline administration produced changes similar to those occurring before saline administration.

**Conclusion:**

The decrease of sinusoidal and increase of cavernosus muscles' EMG activity during micturition apparently denotes sinusoidal relaxation and cavernosus muscles contraction. Sinusoidal muscle relaxation and cavernosus muscles contraction upon micturition are suggested to be mediated through a 'urethro-corporocavernosal reflex'. These sinusoidal and cavernosus muscle changes appear to produce a mild degree of penile tumescence and stretch which might assist in urinary flow during micturition.

## Background

The mechanism of micturition is intricate and under the control of reflex and voluntary actions [1-7]. Various reflexes are involved in facilitating or inhibiting this mechanism [8-11]. The micturition reflex is apparently the main reflex. The urinary bladder, bladder neck, urethra, and external urethral sphincter (EUS) exhibit a well-coordinated behaviour during continuous filling of the urinary bladder [12]. During voluntary voiding, the intravesical pressure increases while the intraurethral pressure decreases and the EUS relaxes.

The penile urethra passes through the corpus spongiosum (CS) which consists of sinusoids. The sinusoids of the corpora cavernosa (CC) and corpus spongiosum (CS) of the penis are contracted and contain a minimal amount of blood in the flaccid phase, while they relax and are full of blood during erection. The bulbo- and ischio-cavernosus muscles (BCM, ICM) contract in the rigid erectile phase [13,14]. The recording of the electric activity of the corporal tissue (CC, CS) was introduced by Wagner and Gerstenberg in 1989 [15] and later studied by other investigators [16-20].

The reaction of the CC, CS or the cavernosus muscles to the passage of urine in the urethra during micturition is not known. We hypothesized that passage of urine in the urethra during micturition stimulates the corporal tissue and cavernosus muscles. This hypothesis was investigated in the current study.

## Methods

### Subjects

Thirty healthy men (mean age 42.8 ± 11.7 SD years, range 29–52) volunteered for the study. They were recruited from our University Hospital workers who were paid. They had no genitourinary complaint in the past or at the time of enrollment. They signed an informed consent after they had been notified about the protocol of the study.

Laboratory work, including urinalysis, blood cell count, liver and kidney function tests as well as electrocardiography showed normal findings. Also, the sonogram of the urinary tracts were normal. The Cairo University Faculty of Medicine Review Board and Ethics Committee approved the study.

### Methods

The subjects were asked to empty their bladders prior to performing the tests. Electromyographic (EMG) electrodes were applied to the penile cavernosus tissue and to the BCM and ICM, and their basal EMG activity was recorded. Full bladder was achieved by asking the subject to continuously drink water until he felt the desire to micturate. The subject was then allowed to micturate. During micturition he was asked to strain, to interrupt micturition and then to evacuate the bladder, while the EMG response of the cavernosus tissue, BCM and ICM was being registered.

The EMG activity was recorded by means of a concentric EMG needle electrode (Type 13 L 49, DISA, Copenhagen, Denmark) measuring 40 mm in length and 0.65 mm in diameter. Two needle electrodes were introduced into each of the corpus cavernosus (CC) and corpus spongiosum (CS): one in the upper and one in the lower third. A ground electrode was applied to the thigh and a strain-gauge respiratory transducer to the thoracic wall. After recording the electric activity, the upper electrode was transferred to the mid third of the CC and the CS, respectively, and the recordings were repeated. The needle electrodes were then transferred to the contralateral CC and the electric activity was recorded.

The EMG activity of the ICM and BCM was recorded by means of a concentric EMG needle electrode (type 13L49, DISA, Copenhagen) measuring 40 mm in length and 0.65 mm in diameter. The ischiopublc ramus of the ICM with the overlying crus penis was palpated and the needle inserted into the ICM lying on its medial aspect. A second identical needle was placed in the BCM; the penile bulb was palpated and the needle electrode introduced into the muscle overlying it.

A standard EMG apparatus (Type MES, Medelec, Woking, UK) was used to amplify and display the recorded potentials. Films of these potentials were taken on a light-sensitive paper (Linagraph type 1895, Kodak, London, UK) from which measurements of the duration of the motor unit action potentials (MUAPs) were obtained. The EMG signals were in addition stored on an FM tape recorder (type 7758A, Hewlett-Packard, Waltham, MA) for further analysis as required. All filtered signals were collected and recorded using an online computer with data acquisition and analysis software (Chart V 4.2, AD instruments, Castle Hill, Sydney, Australia). The acquisition rate was 10 Hz, and the EMG normal band width was 0.1 to 5.0 Hz.

After micturition, erection was induced by electrovibration [21], and the EMG activity of the CC, CS, BCM, and ICM during erection was registered.

### Urethral, CC, CS, BCM, and ICM anesthetization

To examine whether the effect of urethral stimulation, which is produced by the urine passage through the urethra, on the CC, CS, BCM, and the ICM was a direct or reflex effect, the urethra was anesthetized by administration of 5% xylocaine gel (Astra, Södertälje, Sweden). The gel was introduced into the urethra through the gel container nozzle after the urethral orifice had been sterilized by alcohol. Twenty minutes after urethral anesthetization, the EMG activity of the CC, CS, BCM, and ICM was recorded during micturition, on straining during micturition and on micturition interruption. The readings were also taken three hours later when the anesthetic effect had waned. The test was repeated using bland gel instead of xylocaine gel.

On sepate days, each of the BCM, ICM, CC, CP was individually anesthetized and the effect of urine flow through the urethra on these structures was recorded. Two ml of 2% xylocaine were injected into each of the CC, CS, BCM, ICM around the inserted needle electrode; the response of these structures to urine flow through the urethra was then recorded 20 minutes from injection and again 3 hours later. The test was repeated using normal saline instead of xylocaine.

To ensure reproducibility of the results, the recordings were repeated at least twice in the individual subject and the mean value was calculated. The results were analysed statistically using the paired Student's t test, and values were given as the mean ± SD. Significance was ascribed to p < 0.05.

## Results

No adverse side effects were encountered during or after performing the tests and all the subjects were evaluated and the tests completed.

### Electrocavernosogram of the CC and CS

**Figure 1:**
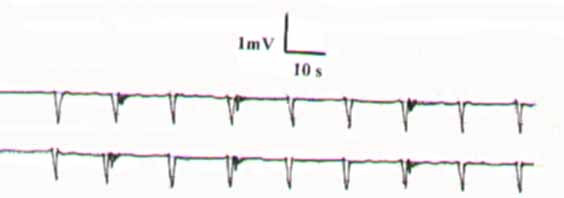
**The basal slow waves and action potentials recorded from the corpus cavernosum.** They have the same frequency, amplitude, and conduction velocity from the two electrodes.

**Figure 2:**
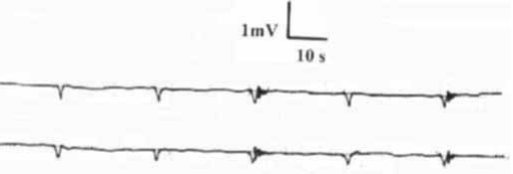
The basal slow waves and action potentials recorded from the corpus spongiosum of the same subject of figure 1.

Basal slow waves (SWs) were recorded from the electrodes applied to the CC and CS (figs. 1, 2). The waves were positive deflected (figs. 1, 2) and had an invariable shape in all the recordings from the same site. In each individual, they exhibited the same frequency, amplitude, and conduction velocity from the 2 electrodes and were constant. The SW variables for the CC and CS are shown in table 1. It is to be observed that the SW variables of the CS exhibited significantly lower values than those of the CC. (figs. 1, 2, table 1). Bursts of fast activity spikes or action potentials (APs) were superimposed on or followed the SWs. They presented as negative deflections and their frequency was inconsistent in each subject (figs. 1, 2).

**Table 1:** The frequency, amplitude, and conduction velocity of the slow waves recorded from the corpora cavernosa (CC) and corpus spongiosum (CS) at rest (basal) and during micturition^+^

**Slow Waves**	**Basal values**				**During micturition**			
	**CC**		**CS**		**CC**		**CS**	
	
	**Mean**	**Range**	**Mean**	**Range**	**Mean**	**Range**	**Mean**	**Range**

**- Frequency (cycle/min)**	4.8 ± 1.3	3.9–6.2	3.6 ± 1.1	2.7–4.8	2.6 ± 0.9*	1.7–3.4	1.2 ± 0.6**	0.9–1.9
**- Amplitude (mV)**	0.58 ± 0.06	0.47–0.77	0.42 ± 0.04	0.31–0.63	0.21 ± 0.03*	0.16–0.34	0.19 ± 0.01**	0.12–0.24
**- Conduction velocity (cm/s)**	5.3 ± 0.9	4.3–6.2	3.6 ± 0.6	3.1–4.4	2.6 ± 0.4*	1.2–3.2	1.4 ± 0.2**	0.9–2.2

+ values were given as the mean ± SD.

* P < 0.05.

** P < 0.01

**Figure 3:**
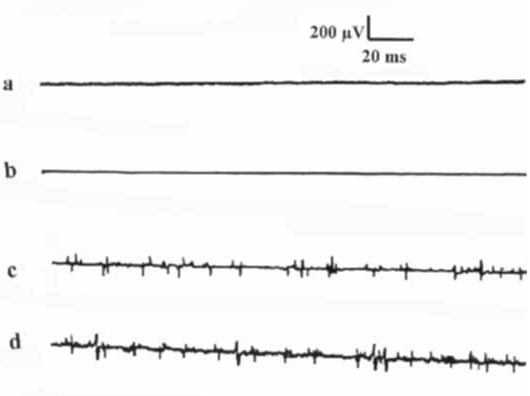
**The EMG activity of (a) bulbocavernosus muscle at rest, (b) ischiocavernosus muscle at rest, (c) bulbocavernosus muscle during micturition, and (d) ischiocavernosus muscle during micturition**.

The BCM and ICM displayed no basal electric activity (fig. 3); no MUAPs were recorded.

**Figure 4:**
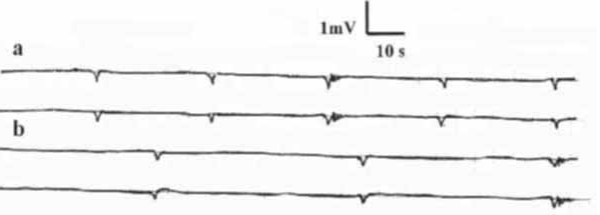
EMG of (A) corpus cavernosum and (B) spongiosum during micturition.

During bladder filling, the EMG activity levels of the CC, CS, BCM, and ICM did not differ from those of the basal ones. However, with the start of micturition, the SW variables of CC and CS decreased, while the MUAPs of BCM and ICM increased (p < 0.05, figs. 3, 4, tables 1, 2). These changes of the wave variables and MUAPs remained constant during the period of micturition. When the urine flow was interrupted, the EMG of the CC, CS, BCM, and ICM returned to the basal values with no significant difference (p > 0.05). When during micturition the subject was asked to strain in an attempt to increase the urine flow, the EMG activity of the CC, CS, BCM, and ICM did not significantly change from the readings without straining (p > 0.05). Upon termination of micturition, the EMG activity of the CC, CS, BCM, and ICM recorded the basal activity (p > 0.05).

**Table 2:** The motor unit action potentials of the bulbocavernosus (BCM) and ischiocavernosus (ICM) muscles before (basal) and during micturition^+^

	**BCM (μV)**		**ICM (μV)**	
	**Mean**	**Range**	**Mean**	**Range**

**Basal**	0	0	0	0
Micturition	76.8 ± 6.4	65–94	69.3 ± 5.8	58–83

+ values were given as the mean ± SD

When rigid penile erection was induced, the EMG of the CC and CS showed a significant reduction (p < 0.01) to a level lower than the one recorded during micturition. Meanwhile, the cavernosal muscles exhibited a significant increase.

### Effect of anesthetization of urethra, corporal tissue and cavernosus muscles

Micturition 20 minutes after anesthetization of the urethra did not produce the significant changes (p > 0.05) in the EMG of the CC, CS, BCM, and ICM which occurred on micturition as already mentioned. Repetition of the test 3 hours after anesthetization, when the anesthetic effect had waned, produced an EMG response similar to that before anesthetization (p > 0.05). Micturition 20 minutes after individual anesthetization of the CC, CS, BCM, and ICM did not effect a significant EMG response in these structures. When the anesthetic effect had waned 3 hours from anesthetization, the EMG response of the CC, CS, BCM, and ICM was similar to that before anesthetization. When we repeated the test after administration of bland gel into the urethra or after injection of saline into the CC, CS, BCM, and the ICM instead of xylocaine, the results were similar to those before gel or saline administration.

The aforementioned results were reproducible with no significant difference when the tests were repeated in the same subject.

## Discussion

In the flaccid phase of erection, the sinusoids contain only a small amount of blood and the smooth muscle fibers are in a state of contraction. In the tumescent stage, the smooth muscle fibers relax and the sinusoids dilate and fill with blood. The smooth muscles surrounding the corporal sinusoids showed in the flaccid phase, a high basal EMG activity indicating that they were in a contracted state [16-20]. Meanwhile, the absence of EMG activity of the cavernosus muscles at rest point to full relaxation of the muscles.

The current study has demonstrated that, during micturition, the urinary flow through the urethra was associated with diminished EMG activity of the smooth muscle fibers of the corporal sinusoids indicating their relaxation. The reduction in the corporal EMG activity was significantly lower than that induced at full erection. Thus, it seems that the passage of urine through the urethra was associated with a small degree of tumescence induced by slight degree of sinusoidal muscles' relaxation. Tumescence disappeared on interruption or termination of micturition as evidenced by return of the EMG activity of the CC and CS to their basal values. The MUAPs of the cavernosus muscles recorded during micturition apparently denote that the muscles are in a state of contractile activity; the recorded EMG activity levels were low compared to those recorded during rigid erection.

The questions that need to be discussed are: what is the effect of the micturition-produced a) mild sinusoidal muscle relaxation and b) mild increase of cavernosus muscles' EMG activity? It appears that the slight sinusoidal relaxation with a resulting mild sinusoidal filling with blood leads to mild penile congestion and stretch. It is known that the penile cavernosus tissue in the flaccid stage is contracted and that the sinusoidal blood volume is scanty. On mild sinusoidal filling with blood during micturition, the penis seems to acquire a degree of tumescence with a resulting mild penile stretch. This penile congestion with stretch seems to effect a degree of penile urethral stretch which might assist in easing the flow of urine through the urethra.

Meanwhile, we do not know the exact role of the mild increase of the EMG activity of the cavernosus muscles during micturition. Does this increase assists in stretching straight the penis by augmenting its blood through tightening the constricting band of the cavernosus muscles situated across the corporal tissue.

Another discussion arising around the process of micturition is whether the response of the CC, CS, BCM, and ICM to urine passing through the urethra is representing a direct or a reflex action. It appeared from our study that the passage of urine through the urethra seems to stimulate the urethral mechanoreceptors which are presumably responsible for the eventually induced effect on the corporal tissue and the cavernosus muscles. This effect is very likely induced through a reflex and not a direct action: the mechanoreceptors seem to transmit the urethral stimuli through the afferent urethral nerves to the spinal cord and then through the efferent nerves to the corporal tissue and cavernosus muscles. The mechanism of action of this assumed reflex needs to be discussed.

### The urethro-corporocavernosal reflex

The EMG response of the corporal tissue and cavernosus muscles to urine traveling through the urethra postulates a reflex relationship between the 2 actions. The constancy of this relationship is indicated by being reproducible. Meanwhile, its reflex nature is evidenced by the absence of the response of the corporal tissue and cavernosus muscles to passage of urine through the urethra upon individual anesthetization of the assumed 2 arms of the reflex arc: the penile urethra as one arm and the corporal tissue/cavernosus muscles as the other arm. We call this reflex relationship the 'urethro-corporocavernosal reflex' (UCCR). It seems necessary to denote that lidocaine does not block the muscle' motor activity but rather the sensory fibers (C and A α-fibers) which are responsible for pain and reflex activity [22,23].

It appears that during micturition the mild sinusoid relaxation and the accompanying mild cavernosus muscles contraction lead to slight penile tumescence and straightening which presumably assist in the passage of the urine through the urethra.

## Conclusion

The decrease of sinusoidal and the increase of cavernosus muscles' EMG activity during micturition apparently denotes sinusoidal relaxation and cavernosus muscles contraction. Sinusoidal muscle relaxation and cavernosus muscles' contraction upon micturition are suggested to be mediated through a 'urethro-corporocavernosal reflex'. These sinusoidal and cavernosal muscles' changes seem to effect a mild degree of penile tumescence and stretch which could assist in urinary flow during micturition. Further studies are warranted to investigate a possible diagnostic significance of this reflex in neurogenic disorders of the urethra or micturition disturbances.

## Abbreviations

corpora cavernosa = (CC), corpus spongiosum = (CS), bulbocavernosus = (BCM) ischiocavernosus = (ICM), electromyographic = (EMG), external urethral sphincter = (EUS) slow waves = (SWs), action potentials = (APs)

## Competing interests

The author(s) declare that they have no competing interests.

## Authors' contributions

AS carried out the study design, data collection, statistical analysis, data interpretation and preparation of manuscript. IA participated in data collection and analysis and literature search. OE participated in data collection, statistical analysis and literature search. AAS participated in data collection and preparation of the manuscript. All authors read and approved the final manuscript.

## Pre-publication history

The pre-publication history for this paper can be accessed here:


